# MR Radiographer/Technologist Practice Issues Surrounding MR Safety

**DOI:** 10.1002/jmri.29508

**Published:** 2024-07-01

**Authors:** Anne Dorte Blankholm, Vera L. Kimbrell, Titti Owman, Sonja Boiteaux, Maureen Hood

**Affiliations:** ^1^ Department of Radiology Aarhus University Hospital Aarhus Denmark; ^2^ Department of Clinical Medicine Aarhus University Aarhus Denmark; ^3^ Forsyth Technical Community College Winston‐Salem North Carolina USA; ^4^ Department of Medical Imaging and Physiology Lund University Lund Sweden; ^5^ Resonant Enterprises, LLC Houston Texas USA; ^6^ Department of Radiology & Radiological Sciences Uniformed Services University of the Health Sciences Bethesda Maryland USA

## Commentary

The MR‐Radiographer/Technologist (MR‐R/T) profession is facing challenging times with the confluence of increasing demand for Magnetic Resonance (MR) Imaging, higher throughput, staff reduction, the emergence of remote scanning, artificial intelligence, and the increasing complexity of cases as more patients have implants. These growing challenges require a renewed focus on developing comprehensive educational, training, and practice guidelines. Development of such guidelines will help MR‐R/Ts to methodically improve state‐of‐the‐art practices.

With MR demand growing around the world, the lack of educational standards, MR safety training, and best practice standards are becoming an international challenge for MR‐R/Ts providing patient care and safety. There are MR safety guidelines, mainly published by the American College of Radiology (ACR), the Royal Australian and New Zealand College of Radiologists (RANZCR), and Medicines and Healthcare Products Regulatory Agency (MHRA).^1^, ^2^, ^3^ These MR safety guidelines, although helpful to MR safety, have omitted or only briefly mentioned key areas of education, training, staffing, and other practice issues that relate directly to patient safety (ACR and MHRA briefly addressing minimum staffing^1^, ^3^). Although current MR practice and MR safety is dictated primarily by physicians, administrators, and physicists, the decisions for standards of education, training, and practice should be made by a team of MR‐R/T leaders and societies collaborating to decide the standards of their practice. Most MR‐R/Ts tend to follow orders rather than applying critical thinking to the processes in their profession.^4^ This partly relates to needing to meet the demands of a busy department. In these types of environments, there may be limited time for reflection on quality improvements, evidence‐based practice, and conducting research projects.^5^ However, an increasing number of MR R/T's is becoming MSc's and PhD's signaling time for a change. MR‐R/Ts are strategically placed to know first‐hand the necessary levels of education, and technical curriculum MR‐R/Ts should possess to practice safely. In addition, it is the working MR‐R/T who comprehends the workflow and staffing combinations needed to improve patient throughput while maintaining safety and patient satisfaction. This document is a call to MR‐R/T leaders around the world to advocate for and participate in the documentation, research, and publication of best practice standards for MR‐R/Ts. Furthermore, international MR‐R/T leaders should establish guidelines for educational levels, specialized training, delineation of duties, responsibilities, and standardization of role descriptors to allow comparison and provide a clearer picture of what is needed globally. Currently, it is extremely difficult to compare safe practice where inconsistent standards are in place.

The ISMRM provided a general international consensus document in 2016 outlining a team incorporating three roles to manage MR safety: 1) MR medical or research director (MRMD/MRRD), 2) MR safety officer (MRSO), and 3) MR safety expert (MRSE).^6^ This document provided an outline, but no specific minimum standard addressing the qualifications for the MRSO, thus allowing a potential safety concern. This gap in MR‐R/T practice and policy highlights the need for MR‐R/T leaders in the field to be more involved in decision making and work to build consensus for curriculum and certification. An example to start accommodating this is the MRSO role descriptor.^7^ Collaboration between societies should be formed to create MR safety educational curriculums for all roles and professions in MR. Currently, activities are ongoing in Europe to collaborate in this regard between the ESR, EFRS, EFMOP, and ESMRMB.

Mainstream MR safety papers have overlooked the safety aspects of adequate staffing in practical terms. Examples are when the MR‐R/T must leave the patient to get the next patient ready, rest room breaks, or when a patient has a medical emergency. In the event of a medical emergency, the solo MR‐R/T must ring for help while getting the patient out of the MR room. Imagine another scenario where the MR‐R/T has a personal medical emergency or gets involved in a projectile accident. Remote scanning also needs to be evaluated as it is now growing but, it remains unclear if this new way to practice adds to safety or increases safety issues. Scenarios like these are extremely challenging for safety, which is why some guidelines note that personnel working in MR should not work alone.^1^, ^3^ This becomes especially troublesome outside of normal department operating hours when staff do work alone. There is also a tendency of mid‐career MR‐R/Ts leaving the workforce while the demand for MR scans is increasing.^8^ Understanding why they are leaving and what today's MR‐R/Ts desire in a meaningful career will guide improvements in MR‐R/Ts practice standards. Additionally, there is a need for MR‐R/T leaders to investigate practice techniques to see which aspects of their practice make a difference to MR‐R/Ts. It is also documented that highly trained and educated MR‐R/T's help to streamline decision making, reduce costs, and improve the quality of care.^9^ MR safety for patients and staff highly depend on culture.^10^ To change clinical practice toward an improved MR safety culture, the MR‐R/T leaders need to be involved and engaged.^10^ Such involvement can empower the MR‐R/T and give a feeling that what they do is important.

Unfortunately, there is very little research into best practices, and what brings better job satisfaction, reduce errors and accidents in MR. Thus, the field of MR needs more evidence‐based research to help guide MR practice. Furthermore, MR‐R/Ts around the world should come together toward an international consensus establishing minimum education, training, and role/responsibility levels for practice of MR‐R/Ts. The diversity of titles and responsibilities from country to country is confusing resulting in the inability to discern proper education and training levels for each of these practice areas. This call to entice MR‐R/Ts to get more involved in research and decision making that guides our profession on standards and will enhance MR safety and increase career satisfaction.
